# Dress Code Monitoring Method in Industrial Scene Based on Improved YOLOv8n and DeepSORT

**DOI:** 10.3390/s24186063

**Published:** 2024-09-19

**Authors:** Jiadong Zou, Tao Song, Songxiao Cao, Bin Zhou, Qing Jiang

**Affiliations:** College of Metrology Measurement and Instrument, China Jiliang University, Hangzhou 310018, China; jiadongzou@cjlu.edu.cn (J.Z.); caosongxiao@cjlu.edu.cn (S.C.); zhoubin@cjlu.edu.cn (B.Z.); jiangq2004@163.com (Q.J.)

**Keywords:** dress code monitoring, YOLOv8n, RFAConv, FLatten, DeepSORT, judgment criterion

## Abstract

Deep learning-based object detection has become a powerful tool in dress code monitoring. However, even state-of-the-art detection models inevitably suffer from false alarms or missed detections, especially when handling small targets such as hats and masks. To overcome these limitations, this paper proposes a novel method for dress code monitoring using an improved YOLOv8n model, the DeepSORT tracking, and a new dress code judgment criterion. We improve the YOLOv8n model through three means: (1) a new neck structure named FPN-PAN-FPN (FPF) is introduced to enhance the model’s feature fusion capability, (2) Receptive-Field Attention convolutional operation (RFAConv) is utilized to better capture the difference in information brought by different positions, and a (3) Focused Linear Attention (FLatten) mechanism is added to expand the model’s receptive field. This improved YOLOv8n model increases mAP while reducing model size. Next, DeepSORT is integrated to obtain instance information across multi-frames. Finally, we adopt a new judgment criterion to conduct real-scene dress code monitoring. The experimental results show that our method effectively identifies instances of dress violations, reduces false alarms, and improves accuracy.

## 1. Introduction

Dress code is a widespread and crucial requirement across various circumstances, including cinemas [1], hospitals [2], kitchens [3], and diverse industrial environments [4]. It serves not only as a regulatory measure for businesses but also as a safeguard for consumers and on-site personnel. However, traditional manual methods of monitoring dress suffer from inefficiency, high cost, and inability to meet the demands of round-the-clock and real-time surveillance.

In recent years, progress in deep learning technologies has offered solutions to these challenges, providing rapid and accurate dress code monitoring at a reasonable cost. One conspicuous example is the YOLO series, among which YOLOv8 [5] has gained particular prominence in edge deployment scenarios. YOLOv8 improves its performance and detection speed by introducing a new backbone network architecture and an anchor-free design. The incorporation of multiscale feature fusion and novel loss functions further contributes to enhanced detection accuracy. Moreover, the scalability of the YOLOv8 model allows for adaptation to various tasks and hardware platforms. This paper proposes a dress code monitoring method tailored to specific industrial scenes based on the YOLOv8 model.

Several studies have utilized deep learning models for dress detection, demonstrating their significant potential for accurately detecting dresses in various scenarios [1,2,3,4,6,7,8,9,10,11,12]. However, some issues have arisen from these studies. Firstly, most of the studies utilize datasets containing over 7000 images, which increases the workload of subsequent manual labeling. Secondly, while these studies emphasize the effectiveness of their proposed object detection models in terms of accuracy, other indicators such as model size and GFLOPs, apart from the FPS, are not fully presented, making the analysis of model performance incomplete. Thirdly, these studies lack actual dress judgment criteria and real-scene testing results. It remains unclear whether these approaches adequately meet practical monitoring requirements. Moreover, due to the complexity of industrial environments and the frequent occurrence of personnel occlusion, even state-of-the-art detection models inevitably suffer from false alarms or missed detections, which make dress judgments based on bounding box-level (bbox-level) detection results unreliable. Therefore, we claim that using only object detection models is insufficient for dress code monitoring tasks, and an additional strategy should be implemented to provide some fault tolerance, which could be achieved through object tracking. With multiple-object tracking (MOT), a unique ID could be assigned to each object. This allows us to utilize the multi-frame information of tracked ID boxes to make comprehensive judgments, thereby alleviating errors caused by single-frame bbox-level judgments. DeepSORT [13] has been demonstrated to be one of the fastest and most robust algorithms for MOT; thus, it was selected as the tracking algorithm for real-time dress monitoring in this study.

Considering the limitations identified in related research, the main contributions of this study are as follows:

1. Introduction of a Lightweight and Efficient Dress Code Monitoring System: We propose a novel real-time dress code monitoring method that relies solely on a small dataset and pursues lightweight implementation. This addresses the challenge of high manual labeling costs associated with large datasets.
2. Improvement of the YOLOv8n Detection Model:
  - We introduce a new architecture named FPN-PAN-FPN (FPF) to improve the model’s feature fusion capability, particularly for scenarios with numerous small targets such as masks and hats.
  - We incorporate the Receptive-Field Attention convolutional operation (RFAConv) [14] into the model network to better capture the differences in information brought by different positions, thereby improving detection accuracy.
  - Inspired by the Vision Transformer, we add the Focused Linear Attention (FLatten) [15] mechanism into the network structure to enhance the model’s ability to capture global contextual information, compensating for the limitations of CNNs in this regard.
3. Comprehensive Evaluation Metrics: Detection performance, tracking performance, and overall dress judgment performance are all presented as comparative indicators in our experiments, providing a more complete analysis of performance compared to previous studies.
4. Proposal of an Instance-Level Judgment Criterion: Leveraging the DeepSORT algorithm, we propose an instance-level judgment criterion and conduct real-scene dress code monitoring, addressing the issue of unreliable bbox-level detection results in complex industrial environments.

The remainder of this paper is structured as follows: Section 2 provides a review of related work, while Section 3 introduces our methodology. In Section 4, we provide a detailed description of the experiment process and analyze the results. Finally, Section 5 summarizes the conclusions.

## 2. Related Work

### 2.1. Review and Challenges of YOLOv8 Framework

**Figure 1:**
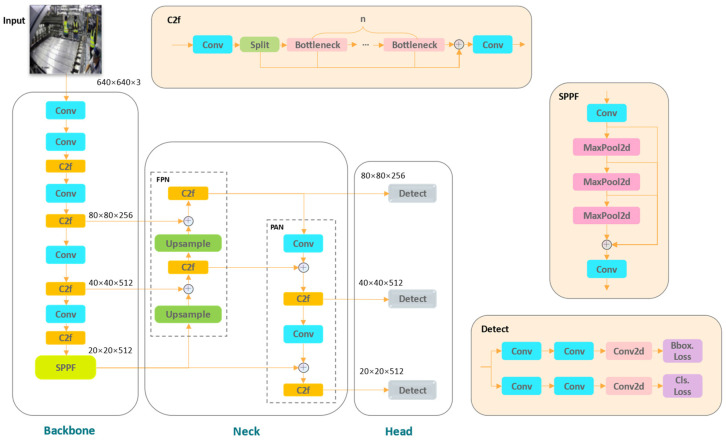
The structure of YOLOv8 network.

YOLOv8 represents a significant advancement in YOLO series, employing an anchor-free detection approach to improve both detection speed and accuracy [16]. Its architecture contains three main components, as delineated in Figure 1: the backbone for initial feature extraction, the neck for feature fusion, and the head for final predictions. Notably, the neck network employs the FPN-PAN architecture, which enhances the entire feature hierarchy with rich semantic information and accurate localization information by up–bottom and bottom–up path augmentation. In the head network, inference occurs across three detection layers sized 80 × 80, 40 × 40, and 20 × 20, demonstrating robust detection capabilities across a wide range of applications.

However, such success could not camouflage the unsatisfactory situation of Small Object Detection (SOD), one of the notoriously challenging tasks in computer vision, owing to the poor visual appearance and noisy representation caused by the intrinsic structure of small targets [17]. Thus, there is still room for improvement in dress detection, particularly in accurately identifying small objects such as hats and masks or distant person targets. To address this issue, the literature [4,18,19] suggests augmenting the existing detection layers with a layer sized 160 × 160. Albeit effective, this approach increases computational complexity while reducing FPS. In this paper, we propose a new neck network as a potential solution to enhance detection performance while preserving efficiency.

### 2.2. Convolutional Innovations in YOLOv8

YOLOv8 extensively utilizes convolutional modules in both the backbone and neck network, as shown in Figure 1. These modules play a critical role in feature extraction, fusion, and parameter optimization. While standard convolutional operations benefit from parameter sharing, thereby reducing parameters and computational complexity, they may fall short in capturing the difference in information brought by different positions, especially for small targets in industrial scenarios.

The introduction of spatial attention mechanisms [20] has led to the development of specialized convolutional operations like CBAMConv and CAConv, derived from Convolutional Block Attention Module (CBAM) [21] and Coordinate Attention (CA) [22], respectively. These mechanisms have proven effective in addressing parameter-sharing issues in convolutional kernels. However, when confronted with large-scale convolutional kernels like 3 × 3 convolution, the weights of the attention map are still shared across each sliding window [14], facing the same dilemma as the convolutional operations in YOLOv8.

Zhang et al. [14] proposed a novel attention mechanism called Receptive-Field Attention (RFA) and its associated convolutional operation RFAConv, which innovatively tackles the parameter-sharing problem by focusing on the receptive-field spatial features. RFAConv provides effective attention weights for large-size convolutional kernels, significantly improving network performance with minimal computational overhead. It makes RFAConv particularly appealing for applications where real-time performance is crucial. Therefore, this novel convolutional operation is introduced in this paper to enhance the model’s ability to capture and process spatial features effectively.

### 2.3. Vision Transformer

The emergence of transformer architectures and self-attention mechanisms has revolutionized various computer vision tasks, including image classification, semantic segmentation, and object detection [23]. A key component in this field is the Focused Linear Attention (FLatten) module [15]. FLatten significantly reduces computational complexity by approximating the Softmax operation, achieving linear complexity while preserving the expressive power necessary for capturing intricate feature relationships in images.

Although other linear attention mechanisms like Performer [24] and Efficient Attention [25] have shown promise in reducing computational overhead, FLatten stands out by addressing the critical aspects of focus ability and feature diversity, essential for object detection tasks. By integrating FLatten into the bottleneck of the C2f module in YOLOv8, we anticipate enhancements in detecting small and salient objects within complex scenes.

### 2.4. Dress Code Detection

Some studies aiming to enhance dress detection performance through the improvement of network structures abound in the literature [1,2,3,4,6,7,8,9,10,11,12]. Lin et al. [4] developed an improved YOLOv8 algorithm, YOLOv8n-SLIM-CA, for safety helmet detection. This model incorporates a CA mechanism and a Slim-Neck structure [26], along with a small target detection layer. It achieved significant improvements over the baseline YOLOv8n, with increases of 2.2% and 3.5% in the mAP0.5 and mAP0.5:0.95 metrics, respectively. Ma et al. [6] proposed YOLO-FL, enhancing YOLOV4-tiny with KIoU and an SPPF module for reflective clothing detection, achieving a mAP of 91.1% and an F1 score of 85%. Its final speed was 116 FPS. Lyu et al. [7] proposed YOLO-CPDC, an algorithm to detect construction personnel’s dress. This algorithm enhances YOLOv5 by introducing Multi-Head Self-Attention into its backbone network, adding a lightweight convolution module (GSA-CBS), and using SIoU in the loss function along with Soft NMS for post-processing. It achieved a mAP0.5 of 93.6% while maintaining a detection speed of 43 FPS. Xiang et al. [27] developed a swift safety helmet detection framework known as FRSHNet, leveraging a multiscale Swin Transformer. It integrates feature extraction through a MAE-NAS backbone, utilizing MobileNetV3’s MobBlock, followed by feature fusion employing a multiscale Swin Transformer module and an efficient-RepGFPN. The network demonstrated superior performance over existing models, achieving high mAP scores on the public datasets.

Additionally, some studies split dress detection tasks into two parts for processing. Lee et al. [28] proposed a two-step CNN-based algorithm for helmet-wearing detection. The algorithm first uses a YOLO model to detect human heads in images and then employs the EfficientNet [29] classification model to categorize the heads into three classes: helmet, head, and hat. It surpassed other models like Faster R-CNN [30], RetinaNet [31], and YOLOv5, achieving an F1 score of 3.2–16.4% higher and obtaining a detection speed of 28 FPS. Jia et al. [32] developed a real-time automatic helmet detection system for motorcyclists in urban traffic using an improved YOLOv5 model. The system is designed in two stages: the first stage detects motorcyclists from video surveillance using YOLOv5-MD (Motorcycle Detection), and the second stage determines whether the motorcyclists are wearing helmets using YOLOv5-HD (helmet detection). The proposed method achieved 97.7% mAP, 92.7% F1 score, and a frame rate of 63 FPS. However, all these studies solely focus on improving detection accuracy without providing actual dress judgment criteria and real-scene testing results. Wei and Yang [33] made dress code judgments based on whether there were helmets and uniforms in the bounding box of the worker according to the detection results of the YOLO model. They still provide no evaluation metrics and real-scene testing results for their criterion. Consequently, it remains unclear whether their approach adequately meets practical monitoring requirements. Moreover, even state-of-the-art detection models inevitably suffer from false alarms or missed detections, which make dress judgments based on bbox-level detection results unreliable. Thus, we claim that using only object detection algorithms for dress code monitoring is insufficient.

Only one study combined YOLO with a multiple object tracking algorithm for safety dress monitoring. Jadhav and Ansari [34] utilized YOLOv4 with DeepSORT to track industrial personnel who were helmet-wearing, triggering an alarm when tracking was lost. However, their description of the method is overly brief and lacks detail. Moreover, the study lacks real-scene experiments, making it difficult to verify its feasibility. Therefore, this paper adopts a combination of object detection and object tracking algorithms to perform instance-level dress code monitoring across multi-frames, incorporating comprehensive judgment criteria and real-scene testing.

### 2.5. DeepSORT Algorithm

Object tracking techniques play an important role in various fields such as industry, agriculture, and transportation [35]. Among the widely used algorithms are the SORT algorithm [36] and the DeepSORT algorithm. SORT combines the Kalman filter [37] and the Hungarian algorithm [38] to predict target states and efficiently associate data among tracked objects, ensuring robust tracking performances.

DeepSORT, an enhancement of SORT, integrates deep learning techniques, particularly convolutional neural networks (CNNs), to extract features from bounding boxes obtained through object detection, a process called Reid (Re-identification). These features are then utilized for object association and trajectory refinement. Additionally, it employs a cascade-matching strategy to associate detected targets with existing trajectories more precisely while swiftly eliminating poor matches in the early stages, thus reducing unnecessary computational overhead. DeepSORT outperforms SORT, especially in scenarios with significant appearance variations and occlusions [13], achieving a running speed of 31 FPS when integrated with the YOLO model and deployed on the Jetson Xavier NX device [39]. Due to its real-time tracking capability and robustness, DeepSORT was chosen as the tracking algorithm in this study.

## 3. Methodologies

### 3.1. Objectives

To comprehensively evaluate our methodology, we will assess it in three dimensions: object detection, object tracking, and overall judgment performance. The specific objectives are as follows:

1. Maximize the accuracy of the object detection model while minimizing model size and computational overhead given the hardware and dataset size;
2. The tracking algorithm must provide stable and real-time instance ID information as a foundation for subsequent dress code judgments;
3. Combined with the judgment criterion, our proposed method must effectively identify instances of dress violations while minimizing false alarms and maximizing accuracy during real-scene video testing.

### 3.2. Overall Framework

**Figure 2:**
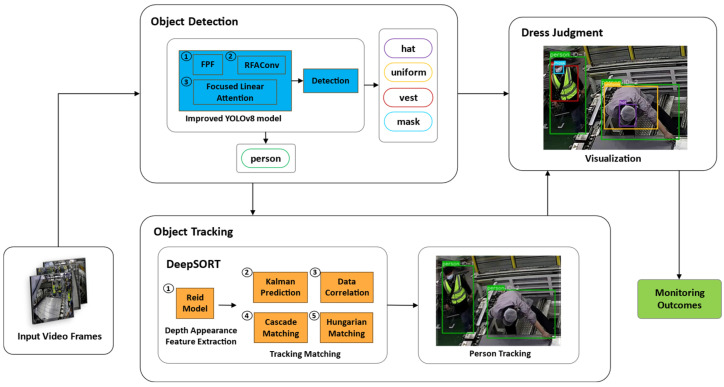
Overall framework of our dress code monitoring method.

Figure 2 depicts the overall framework we developed for dress code monitoring. This framework contains an improved YOLOv8 model, an object tracking algorithm, and a dress judgment criterion. Initially, video frames are input into the improved YOLOv8 model to locate dress-related objects. Subsequently, the location information of the person class and the current frame are fed into the DeepSORT tracking model to derive a unique ID. Through the integration of output from both object detection and tracking processes, we make an instance-level dress judgment across multi-frames, ultimately yielding the monitoring outcomes.

### 3.3. Improved YOLOv8n

**Figure 3:**
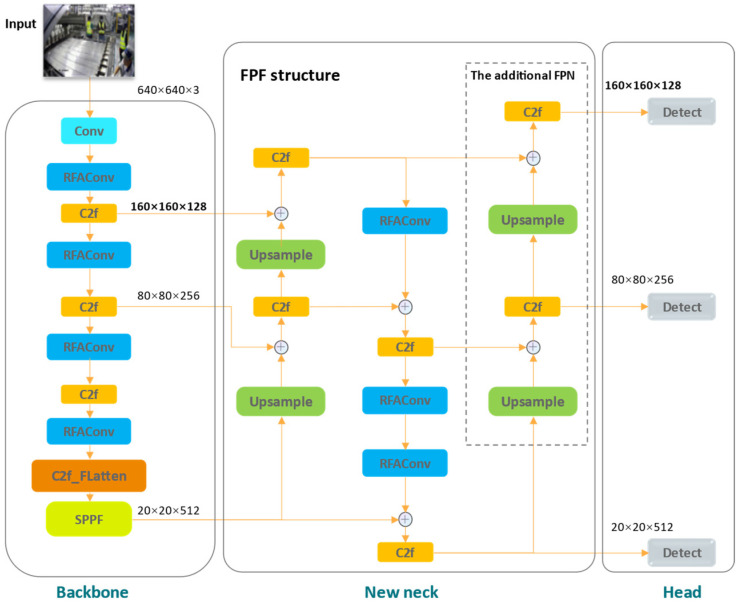
Improved YOLOv8 network. The original neck structure is replaced by the proposed FPF structure. RFAConv replaces all standard convolutions in the network except for the first one, and the Flatten module is added before the SPPF layer.

To improve detection accuracy, we propose an improved YOLOv8 model for dress detection. Specifically, we designed a new neck network called the FPF structure. After that, we incorporated the RFAConv, depicted in deep blue, replacing all original convolutional modules in the network except for the first one. Then, we added the FLatten mechanism into the bottleneck of the C2f module, just before the SPPF structure, shown in deep orange. The overall structure of the improved YOLOv8 is depicted in Figure 3.

#### 3.3.1. Introduction of the FPF Structure

**Figure 4:**
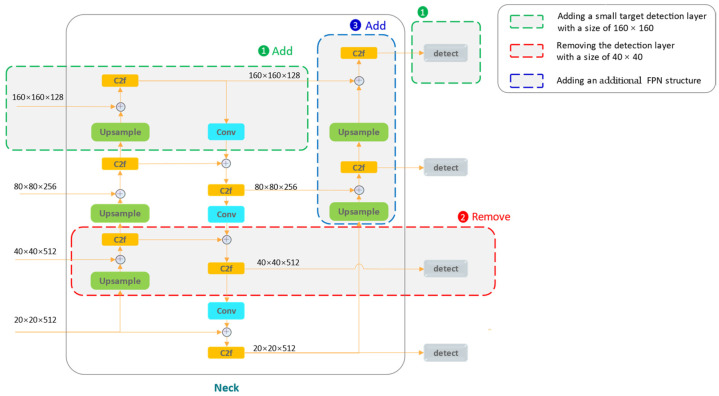
Development details of the FPF structure. The design process is divided into three key steps, as shown from Step 1 to Step 3.

Figure 4 illustrates the development details of the FPF structure. We follow the approach of incorporating a small target detection layer sized 160 × 160, prompting us to contemplate the issue of redundant detection layers. Considering the diverse and significantly varying scales of detection objects in industrial scenes, as shown in a figure in Section 4.1.1, we remove the detection layer sized 40 × 40 along with the corresponding horizontally connected scale feature maps in the neck network. It is well known that the detection layer sized 20 × 20 is well-suited for predicting large objects, while the detection layer sized 80 × 80 is adept at predicting medium objects; thus, we choose to retain both.

Undoubtedly, the removal of the detection layer sized 40 × 40 will, to some extent, reduce the model size and computational complexity, but it may impact detection accuracy. As a remedy, we introduce an additional FPN after the FPN-PAN structure in the neck network, forming the FPF structure. This newly added FPN operates up–bottom, allowing high-level semantic information to be passed down, thereby further enhancing the localization information with deep features. Moreover, when predicting small targets in the final head section, the FPF structure performs predictions on three types of feature maps sized 160 × 160, which are concatenated along the channel dimension. Previous methods [4,16,17] only concatenated two types of small target detection layers, thus our structure could show better performance for Small Object Detection (SOD). Additionally, the FPF structure enriches the model’s feature hierarchy with tridirectional paths, further enhancing the feature fusion capability.

#### 3.3.2. Incorporation of the RFAConv

RFAConv [14] is a novel approach that dynamically generates attention weights for each receptive field within the network. This mechanism effectively addresses the parameter-sharing issue prevalent in standard convolutional kernels, allowing for a more refined and adaptive feature extraction process. By emphasizing the significance of different features within the receptive field, the RFAConv enables the network to discern and focus on the most informative aspects of the input data, thus improving the detection accuracy for objects of various sizes and shapes.

**Figure 5:**
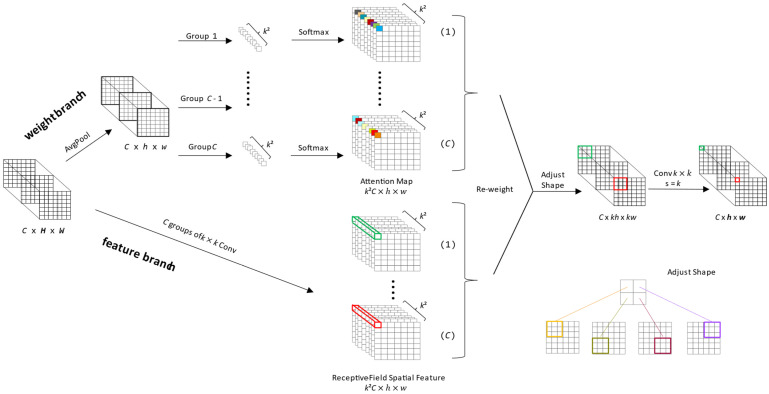
The detailed structure of RFAConv [14]. The input feature map is processed through two branches to obtain attention maps and receptive-field spatial feature maps, which are then re-weighted and adjusted to achieve feature extraction with non-shared parameters.

As depicted in Figure 5, *C*, *H*, and *W* represent the number of channels, height, and width of the input feature map. RFAConv employs two distinct branches to obtain attention maps and receptive-field spatial feature maps. In the weight branch, average pooling is employed to aggregate global information for each input feature. Then, *C* groups of 1 × 1 convolutions are used to facilitate information interaction, while effectively avoiding feature overlap when extracting features via sliding windows. After that, Softmax emphasizes the importance of each feature in the receptive-field feature, resulting in *C* groups of attention maps with a shape of *k*^2^ × *h* × *w*, where *k* represents the size of the original standard convolutional kernel such as 3. In the feature branch, the input feature map is processed through *C* groups of convolutions with size of *k* × *k* to extract features, similarly yielding *C* groups of spatial feature maps with a shape of *k*^2^ × *h* × *w*. The outcomes of two branches are then subjected to multiplication (i.e., Re-weight) and shape adjustment operations, ultimately producing receptive field spatial feature maps with a shape of *C* × *kh* × *kw*. The calculation process can be expressed as shown in Equation (1). Subsequently, standard convolutional kernels with size of *k* × *k* and strides of *k* can be employed to achieve feature extraction with non-shared parameters.

(1) $$\begin{array}{l} F=\mathrm{Softmax}\left(g^{1\times 1}\left(\mathrm{AvgPool}\left(X\right)\right)\right)\times \;\mathrm{ReLU}\left(\mathrm{Norm}\left(g^{k\times k}\left(X\right)\right)\right)\\ \;\;=A_{\mathrm{rf}}\;\times {\;F}_{\mathrm{rf}}.\; \end{array}$$

$g^{i\times i}$ represents a group convolution of size i×i, *k* represents the size of the convolutional kernel, *Norm* stands for normalization, *X* represents the input feature maps, and *F* is obtained by multiplying the attention map $A_{rf}$ with the transformed receptive-field spatial feature $F_{rf}$ [14].

In this paper, considering the extensive use of RFAConv could prolong the processing time for each image, we replace all standard convolutional layers in the YOLOv8 network architecture with RFAConv, except for the first one, as shown in Figure 3. Our preliminary experimental results show that continuing to replace the first standard convolutional layer with RFAConv reduces the FPS by 25 and increases the GFLOPS by 0.2 compared to our improved model. Moreover, this modification does not yield a discernible improvement in accuracy of our final detection model, and it significantly extends the model’s training time. Therefore, we retain the first standard convolutional layer, which processes the original image with the largest scale (640 × 640).

#### 3.3.3. Addition of the Focused Linear Attention

Building upon the transformative impact of transformer architectures in computer vision tasks as discussed in our related work section, we add the Focused Linear Attention (FLatten) module into the YOLOv8 architecture. The FLatten module is recognized for its efficiency in computational reduction by approximating the Softmax operation, a critical step towards managing the complexity of high-dimensional visual data. Its performance enhancement primarily stems from two aspects: improving focus ability and increasing feature diversity.

The FLatten module enhances the model’s focus ability by adjusting the feature directions of the query and key vectors through a novel focused function $f_{p}\left(x\right)$. This adjustment sharpens the model’s attention on the most informative features, crucial for accurate object detection. Additionally, the depthwise convolution (DWC) within FLatten serves to restore the rank of the attention matrix, ensuring diversity in feature representation. This rank restoration is vital for maintaining the richness of the feature set, which is often compromised in traditional linear attention mechanisms. Specifically, the module can be formulated as follows:

(2) $$\begin{array}{c} O=\mathrm{Sim}\left(Q,K\right)V=\emptyset_{p}\left(Q\right)\emptyset_{p}{\left(K\right)}^{T}V+\mathrm{DWC}\left(V\right),\\ \mathrm{where}\;\emptyset_{p}\left(x\right)=f_{p}\left(\mathrm{ReLU}\left(x\right)\right),{\;f}_{p}\left(x\right)=\frac{\left\|x\right\|}{\left\|x^{**p}\right\|}x^{**p}, \end{array}$$

and $x^{**p}$ represents element-wise power p of x. *O* represents the output of the attention module, *Q* and *K* are the query and key matrices, respectively, and *V* is the value matrix. The function $\emptyset_{p}$ is derived from the focused function $f_{p}\left(x\right)$, which is applied to the query and key vectors to adjust their feature directions and enhance the model’s focus ability. The term *DWC*(*V*) denotes the depthwise convolution applied to the value matrix, which serves to restore the matrix rank and maintain feature diversity. This formulation ensures that while the model’s complexity remains linear, its expressive power is preserved, if not enhanced.

The FLatten module improves the ability to capture global contextual information, allowing the model to further focus on critical and sensitive areas, particularly in regions of the image where personnel frequently operate. In this paper, we choose to insert the module into the bottleneck of the C2f module, just before the SPPF. This location is selected for its feature map scale of 20 × 20, which means that the addition of the FLatten module can effectively capture fine-grained details without incurring excessive computational overhead. Its size is also at a stage where the network begins to aggregate information from high layers, making it an optimal location to enhance the model’s ability to focus on informative features. By adding the FLatten, we expect significant improvements in YOLOv8’s performance, particularly in detecting small and salient objects within complex industrial scenes. The module’s ability to focus on informative features and maintain feature diversity is anticipated to refine the model’s accuracy and robustness in various object detection scenarios.

### 3.4. Dress Code Judgment Criterion Using DeepSORT

We employ the improved YOLOv8n to identify hat, mask, uniform, vest, and person classes. The location information of the person class and the current frame are then fed into the DeepSORT algorithm, which is responsible for the person’s trajectory and maintains a tracking bounding box with a unique ID. Dress code judgment is conducted based on whether the required dresses are contained within this tracking box. Specifically, we set the tracking frame cycle for each instance’s dress judgment as G, and the threshold number of hazardous boxes as T. From the start of tracking, if the current tracked person is not wearing the required dresses completely, this tracking box is marked as a hazardous box. We keep a count of hazardous boxes for each tracking ID. In each sequence of tracked G frames, if the number of hazardous boxes reaches T, the tracked person is deemed to violate dress regulations, triggering an alarm and recording the violation. The hazardous box count is then reset, and the next G frame cycle begins, continuing this process in a loop. By incorporating predictions from multi-frames to make an instance-level dress judgment, the model’s misjudgment, which are largely caused by dress deformation and occlusion due to various human poses such as turning and squatting, could be significantly alleviated.

It is important to note that the performance of this dress code judgment criterion relies heavily on a high-precision object detection algorithm and a tracking algorithm capable of stable person tracking in this industrial scene. Therefore, in the following experiment section, we will conduct performance evaluations of the object detection and tracking algorithms to ensure that this dress code judgment criterion can meet the practical needs of real-scene industrial testing.

**Figure 6:**
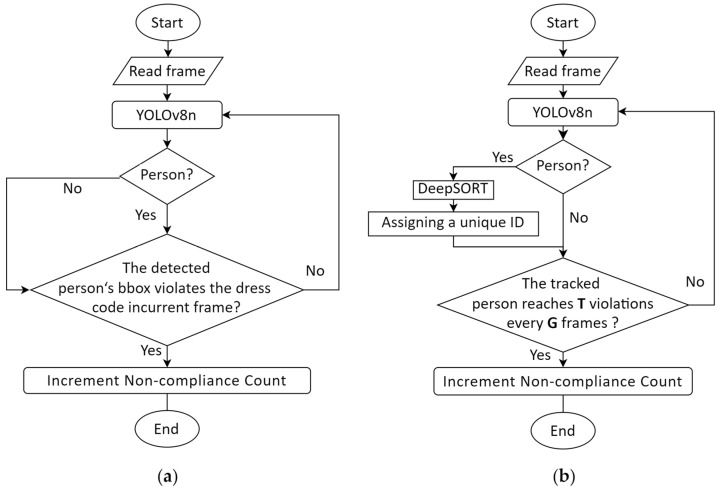
Flowchart of two dress code judgment criteria. (**a**) Bbox-level judgment criterion; (**b**) instance-level judgment criterion. The bbox-level method makes judgments based on whether the required dresses are present in the currently detected person’s bounding box. The instance-level method builds upon the bbox-level method by integrating multi-frame detection results with unique IDs for comprehensive evaluation. G: the tracking frame cycle for each instance’s dress judgment; T: the threshold number of hazardous boxes.

Additionally, to further demonstrate the effectiveness of our method, we compare it with the traditional bbox-level approach, which solely depends on the detection outcomes from the YOLOv8n. The bbox-level approach makes judgments based on whether the required dresses are present in the currently detected person’s bounding box. Refer to Figure 6 for specific judgment criteria. We believe that integrating object detection with tracking could greatly enhance the fault tolerance of dress monitoring tasks.

## 4. Experiments and Analysis

### 4.1. Experimental Settings

#### 4.1.1. Dataset

**Figure 7:**
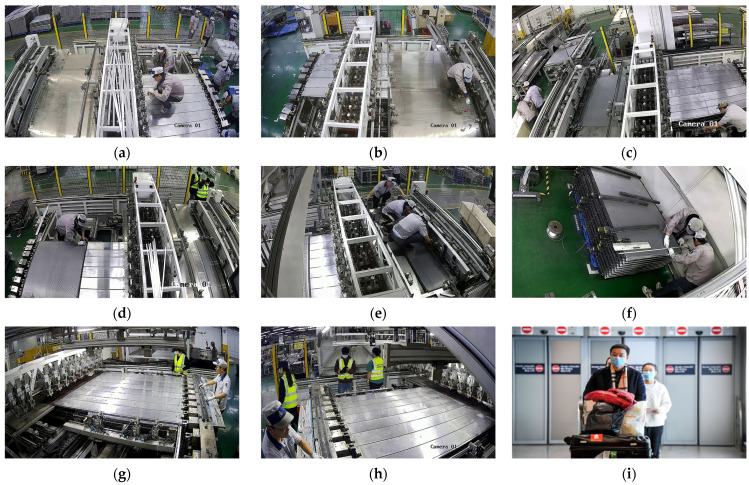
Some representative images in our Dress Dataset. (**a**–**h**) Monitoring scenes from one to eight; (**i**) a representative image from the Mask Dataset.

For object detection, we construct a specialized Dress Dataset related to this industrial scene, which contains five different classes of objects, including hat, mask, uniform, vest, and person. The majority of this dataset is collected from eight surveillance cameras in this industrial scene, with a resolution of 1280 × 720. The dataset spans over a month, and the data collection method is to save one frame from the surveillance footage every 30 s, followed by manual selection. This ensures the diversity and generalization of the dataset, as it includes various lighting conditions and different personnel at work. Additionally, considering that workers in these scenes often perform tasks with their heads down, resulting in fewer instances of the mask class being captured, we supplement our dataset by leveraging the Mask Dataset [40]. To adapt the Mask Dataset to our Dress Dataset, we choose a portion of its images and adjust their notations, ultimately increasing the adequate number of mask class. Some representative images in our Dress Dataset are shown in Figure 7.

**Figure 8:**
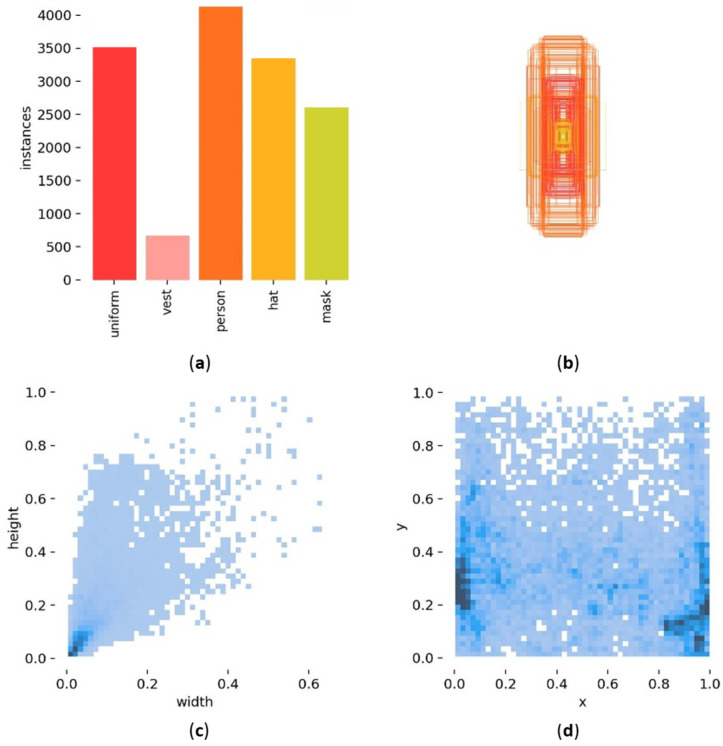
Statistical information about the objects in Dress Dataset. (**a**) Bar chart of the quantity of all classes; (**b**) size distribution of the object bounding boxes; (**c**) scatter plot of widths and heights of bounding boxes; (**d**) spatial distribution of object positions.

Figure 8 shows the information related to the manual labeling of the objects in this dataset. Specifically, Figure 8a displays the quantity of objects for each class in the dataset, indicating that the quantities are generally balanced except for the vest class. Despite this imbalance, the model’s excellent detection performance on the vest class is noteworthy, allowing us to overlook this disparity. This can be attributed to the significant color contrast of the vest class compared to other classes, facilitating effective feature extraction. This strategy also aligns with our original intention to meet industrial requirements through the implementation of deep learning techniques based on limited samples.

Figure 8b illustrates the sizes of bounding boxes corresponding to all classes in the dataset, revealing the presence of objects across various scales, spanning a wide range of sizes. Figure 8c depicts a scatter plot showcasing the widths and heights of object bounding boxes, with the deepest color concentration observed in the bottom-left corner of the plot, which suggests that the dataset contains a large number of small objects. This explains why we introduce the detection layer with a size of 160 × 160 to enhance the model’s accuracy in detecting small objects.

Figure 8d depicts the distribution of object bounding box center points, revealing that the centers of objects are mainly concentrated in the bottom-left and bottom-right regions of the image area. This further elucidates the rationale behind our incorporation of RFAConv to capture the differences in information brought by different positions.

This paper divides the Dress Dataset into two parts: a training set comprising 2731 images, which includes 2440 images from industrial scenes and 291 images from Mask Dataset, and a testing set consisting of 606 images from industrial scenes.

For the performance evaluation of the object tracking algorithm, we select four representative real-scene video segments with a total of 1250 frames from these industrial sites, which includes scenarios with multiple overlapping, varying distances of targets, and other complex situations.

For the performance evaluation of the overall dress judgment, we use videos that are not included in the model training phase. These videos, obtained from multiple on-site surveillance cameras, total approximately 4 min and comprise 5750 frames, encompassing various lighting conditions and different personnel.

#### 4.1.2. Implementation

The operating system used in this study is Ubuntu 22.04. The hardware configuration includes an Intel(R) Core(TM) i7-12700 CPU with 32 GB of RAM and an NVIDIA GeForce RTX 3060 GPU with 12 GB of VRAM. The programming language is Python 3.8, the deep learning framework is PyTorch 2.2.1, and the CUDA version is 12.1. Furthermore, given our emphasis on real-time operation and lightweight design, we select YOLOv8n as the baseline model. To mitigate overfitting, this experiment employs several data augmentation methods to enhance the self-made dataset and we set the patience value to 30. For our training process, some key parameters utilized are as follows:

- Input image size: 640 × 640;
- Epoch: 250;
- Initial learning rate: 0.01;
- Weight decay: 0.0005;
- Batch size: 8;
- Optimizer: SGD;
- Patience: 30;
- Data enhancement strategy: Mosaic, flip, scale, translate, hsv.

### 4.2. Evaluation Metrics

#### 4.2.1. Evaluation Metrics for Object Detection

In our study, we evaluate the detection performance of our improved YOLOv8n model using various evaluation metrics, including ${\mathrm{precision}}_{\mathrm{Det}},\;{r\mathrm{ecall}}_{\mathrm{Det}},{\mathrm{mAP}}_{\mathrm{Det}}0.5,\;{\mathrm{mAP}}_{\mathrm{Det}}0.5:0.95$, model size, GFLOPs, and FPS, where the Det stands for dress detection. These metrics are calculated based on the following parameters: true positives (${\mathrm{TP}}_{\mathrm{Det}}$), false positives (${\mathrm{FP}}_{\mathrm{Det}}$), and false negatives (${\mathrm{FN}}_{\mathrm{Det}}$).

${\mathrm{Precision}}_{\mathrm{Det}}$ is the ratio of the number of ${\mathrm{TP}}_{\mathrm{Det}}$ predicted by the model to the total number of positive predictions detected. ${\mathrm{Recall}}_{\mathrm{Det}}$ is the ratio of the number of ${\mathrm{TP}}_{\mathrm{Det}}$ correctly predicted by the model to the total number of actual positive samples. ${\mathrm{AP}}_{\mathrm{Det}}$ represents the average precision of the model at various recall rates, calculated as the area under the precision–recall curve. The mean average precision (${\mathrm{mAP}}_{\mathrm{Det}}$) is calculated as the average of the ${\mathrm{AP}}_{\mathrm{Det}}$ values across all sample classes. It serves as a metric to evaluate the model’s detection performance across all classes.

#### 4.2.2. Evaluation Metrics for Object Tracking

For object tracking, we employ the current mainstream evaluation metrics MOTP and MOTA [41]. MOTP measures how precisely a tracking algorithm can locate objects in the tracking frame. Its calculation formula is as follows.

(3) $$\mathrm{MOTP}=\frac{\sum_{i,t}d_{t,i}}{\sum_{t}C_{t}},$$

where $d_{t,i}$ represents the bounding box overlap of target i with its assigned ground truth object at frame t and $C_{t}$ represents the number of successfully matched objects at frame t.

MOTA measures the overall performance of the tracker in the tracking task, including the number of detected objects, tracking accuracy, and the consistency of object identities.

(4) $$\mathrm{MOTA}=1-\frac{\sum_{t}\left({\mathrm{FN}}_{t}+{\mathrm{FP}}_{t\;}+{\mathrm{IDSW}}_{t}\right)}{\sum_{t}g_{t}}\;,$$

where ${\mathrm{FN}}_{t}$ is the number of ground truth objects missed by the tracker at frame t, ${\mathrm{FP}}_{t}$ is the number of false positives made by the tracker at frame t, ${\mathrm{IDSW}}_{t}$ is the number of ID switches occurring at frame t, and $g_{t}$ is the total number of ground truth objects at frame t.

#### 4.2.3. Evaluation Metrics for Dress Code Monitoring

To evaluate our dress code monitoring method, we modified conventional evaluation metrics to adapt to this specific application scenario. Specifically, these metrics encompass ${\mathrm{precision}}_{\mathrm{DC}}$, ${\mathrm{recall}}_{\mathrm{DC}}$, and ${\mathrm{accuracy}}_{\mathrm{DC}}$ and we use the subscript DC to denote metrics associated with dress code monitoring. Table 1 shows the detailed definitions.

${\mathrm{Precision}}_{\mathrm{DC}}$ is the ratio of the number of tracking boxes correctly predicted as non-compliance by the model to the total number of tracking boxes predicted as non-compliance.

(5) $${\mathrm{Precision}}_{\mathrm{DC}}=\frac{{\mathrm{TP}}_{\mathrm{DC}}}{{\mathrm{TP}}_{\mathrm{DC}}+{\mathrm{FP}}_{\mathrm{DC}}}\;.$$

${\mathrm{Recall}}_{\mathrm{DC}}$ is the ratio of the number of tracking boxes correctly predicted as non-compliance by the model to the total number of tracking boxes that are actually non-compliance.

(6) $${\mathrm{Recall}}_{\mathrm{DC}}=\frac{{\mathrm{TP}}_{\mathrm{DC}}}{{\mathrm{TP}}_{\mathrm{DC}}+{\;\mathrm{FN}}_{\mathrm{DC}}}\;.$$

${\mathrm{Accuracy}}_{\mathrm{DC}}$ is the ratio of the number of tracking boxes where the model correctly predicts compliance or non-compliance with dress code to the total number of tracking boxes.

(7) $${\mathrm{Accuracy}}_{\mathrm{DC}}=\frac{{\mathrm{TP}}_{\mathrm{DC}}+{\mathrm{TN}}_{\mathrm{DC}}}{{\mathrm{TP}}_{\mathrm{DC}}+{\mathrm{FP}}_{\mathrm{DC}}+{\mathrm{TN}}_{\mathrm{DC}}+{\;\mathrm{FN}}_{\mathrm{DC}}}\;.$$

### 4.3. Experiments on Detection

#### 4.3.1. Comparation of YOLOv8n, YOLOv8n-P2, and YOLOv8n-FPF

To verify the effectiveness of the proposed FPF structure, we conduct comparative experiments on YOLOv8n, YOLOv8n-P2 (i.e., adding an extra detection layer with a size of 160 × 160), and YOLOv8n-FPF (i.e., using FPF structure), while maintaining consistent training conditions across all experiments. The results shown in Table 2 demonstrate that adding an extra detection layer to the YOLOv8n network indeed improves the model’s ${\mathrm{mAP}}_{\mathrm{Det}}0.5$ and ${\mathrm{mAP}}_{\mathrm{Det}}0.5:0.95$ by 2.8% and 2.6%, respectively. However, this strategy also increases the computational overhead and complexity of the model, as evidenced by the increase in model size, GFLOPs, and the decrease in FPS.

In contrast, adopting the FPF structure not only achieves superior detection accuracy but also achieves nearly identical detection speed compared to YOLOv8n-P2, with lower GFLOPs and model size due to the removal of the detection layer sized 40 × 40. The model size even falls below that of the original YOLOv8n. This strongly confirms the effectiveness of introducing the FPF structure in this application scenario.

#### 4.3.2. Optimal Location for Inserting the FLatten Module

**Figure 9:**
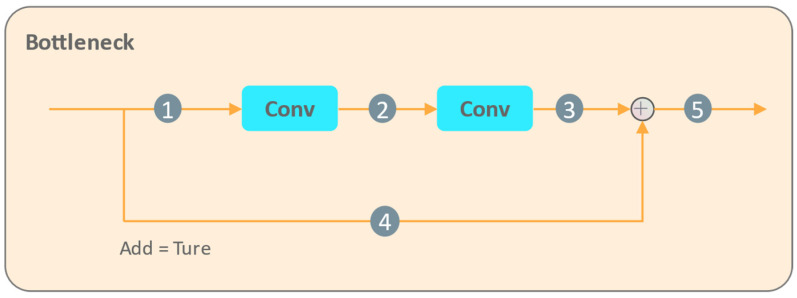
Different potential locations for inserting the FLatten module in the C2f bottleneck.

After introducing the FPF structure and RFAConv, we explore the optimal location for inserting the FLatten module among five potential positions. “Location1” indicates that the FLatten module is inserted at position 1 in Figure 9, and so on. According to the experimental results in Table 3, the best detection performance is achieved when the FLatten module is inserted at position 3. Compared with the FLatten-free model, ${\mathrm{mAP}}_{\mathrm{Det}}0.5$ and ${\mathrm{mAP}}_{\mathrm{Det}}0.5:0.95$ are improved by 0.1% and 0.4%, respectively. This means that at this position, the FLatten module can directly leverage the high-level features obtained after two convolutions and non-linear transformations. It further enhances the selection and weighting of important features, thereby improving the overall feature representation capability and model performance.

#### 4.3.3. Overall Comparison with YOLOv8n

**Figure 10:**
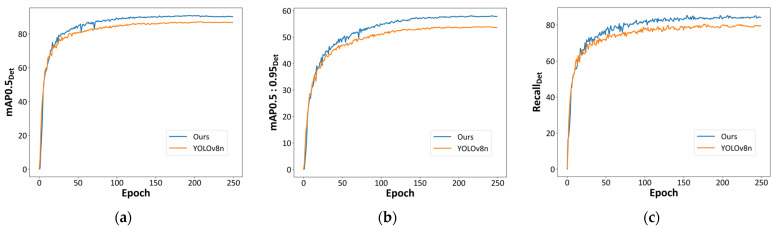
Comparison of ${\mathrm{mAP}}_{\mathrm{Det}}$ and ${\mathrm{Recall}}_{\mathrm{Det}}$ between the original YOLOv8n and our model. (**a**) ${\mathrm{mAP}}_{\mathrm{Det}}0.5$, (**b**) ${\mathrm{mAP}}_{\mathrm{Det}}0.5:0.95$, and (**c**) ${R\mathrm{ecall}}_{\mathrm{Det}}$.

Figure 10 depicts the change curves of some important evaluation metrics for the original YOLOv8n and our model throughout the training process. From the figure, it is clear that our proposed model outperforms YOLOv8n in three detection metrics: ${\mathrm{mAP}}_{\mathrm{Det}}0.5,\;{\mathrm{mAP}}_{\mathrm{Det}}0.5:0.95$, and ${r\mathrm{ecall}}_{\mathrm{Det}}$ after approximately 20 epochs of training. Additionally, our model begins to stabilize after about 150 epochs of training. Compared with the baseline, our model achieves superior detection performance with fewer missed detections.

Table 4 shows the ${\mathrm{AP}}_{\mathrm{Det}}$ values for each dress class and the ${\mathrm{mAP}}_{\mathrm{Det}}$0.5 values for all classes for the original YOLOv8n and our model. It is clear that the ${\mathrm{mAP}}_{\mathrm{Det}}$0.5 value of our model has improved by 3.9%. Especially noteworthy are the AP values for the hat and mask classes, which have increased by more than 8%. This indicates that our improved model can effectively increase the detection accuracy of small objects and improve the detection performance.

#### 4.3.4. Comparison with Other Detection Models

To further demonstrate the effectiveness of our improved model, we conducted the following comparison experiments involving various mainstream models. All of the experiments were conducted using the Dress Dataset with the same training parameters.

According to the comparative experimental results shown in Table 5, the classic two-stage detection algorithm Faster R-CNN [30] and the one-stage detection algorithm SSD [42] comprehensively underperform when compared to the YOLO series in terms of detection performance. Moreover, their substantial computational overhead and low real-time performance render them completely inadequate for this application scenario. In the YOLOv5 [43] series, YOLOv5n stands out as the most lightweight model, having the smallest model size, lowest computational overhead, and fastest detection speed (294FPS). However, its lower ${mAP}_{Det}$ values pose challenges for accurately detecting various classes. While YOLOv5s and YOLOv7-tiny [44] demonstrate similar and excellent detection performance across various evaluation metrics, they fall short in terms of model size.

YOLOv8n and YOLOv8s [5], as two different scaled versions within the advanced YOLO series, outperform models of the same scale in the YOLOv5 series in terms of detection performance. However, their original three detection layers are insufficient for tasks requiring identifying numerous small targets.

Several recently proposed algorithms, such as YOLOv9t [45], YOLOv10n [46], and GOLD-YOLO [47], have shown poor detection performance in this industrial scenario. Although RT-DETR-Resnet18 [48] excels in terms of ${\mathrm{mAP}}_{\mathrm{Det}}$, it still fails to meet the lightweight requirements. Additionally, its low FPS raises concerns about its ability to meet real-time monitoring demands when integrated with the tracking algorithm later.

In the results of our comparative experiments, our improved model achieves the highest ${\mathrm{mAP}}_{\mathrm{Det}}0.5$ and ${\mathrm{mAP}}_{\mathrm{Det}}0.5:0.95$ values, even surpassing that of RT-DETR-Resnet18. Additionally, it maintains a very small model size, along with reasonable GFLOPs. While this to some extent sacrifices detection speed, its overall detection performance remains superior to that of other models.

#### 4.3.5. Ablation Study

To verify the effectiveness of each improvement strategy proposed in this paper, we perform ablation experiments on the baseline model using the Dress Dataset.

As shown in Table 6, the substitution of the neck section of the baseline with FPF structure significantly enhances the detection performance in our scenario while only sacrificing a small portion of GFLOPs and FPS. This demonstrates that the FPF structure designed in this paper provides better localization and classification capabilities for objects across various scales, especially for small targets. RFAConv effectively captures subtle differences in information brought by various positions using receptive-field spatial features. When compared to the baseline model, the ${\mathrm{mAP}}_{\mathrm{Det}}0.5$ and ${\mathrm{mAP}}_{\mathrm{Det}}0.5:0.95$ have increased by 0.8% and 1.3%, respectively. The addition of the FLatten module expands the receptive field to a larger region while maintaining the nearly same computational overhead, enjoying the advantage of modeling long-range dependencies. This allows the model to focus more on areas where personnel frequently operate. The ${\mathrm{mAP}}_{\mathrm{Det}}0.5$ and ${\mathrm{mAP}}_{\mathrm{Det}}0.5:0.95$ were improved by 0.8% and 0.7%, respectively.

The last row showcases the final results of our dress detection model, demonstrating an increase of 3.9% and 4.3% in ${\mathrm{mAP}}_{\mathrm{Det}}0.5$ and ${\mathrm{mAP}}_{\mathrm{Det}}0.5:0.95$, respectively. Additionally, we observe that the model size is even lower than those of baseline, while FPS and GFLOPs remain within a reasonable range, aligning with the requirements for lightweight model. These findings indicate that the integration of these modules into YOLOv8n has produced positive results: the FPF structure enhances the detection of small objects, the RFAConv module captures more detailed information during feature extraction and highlights key regions in the input data, while the FLatten module improves the ability to capture global contextual information, allowing the model to further focus on critical and sensitive areas.

These experimental findings emphasize that our enhancements to YOLOv8n have effectively enhanced the accuracy of object detection.

#### 4.3.6. Visualization Analysis

Deep learning models are often questioned for their lack of interpretability. To visually illustrate the detection capability of our improved model, we utilize the Grad-CAM (Gradient-weighted Class Activation Mapping) [49] technique for visual analysis. Grad-CAM generates visual explanations for the decisions made by deep neural networks, highlighting the regions in an image that are critical for the model’s predictions.

**Figure 11:**
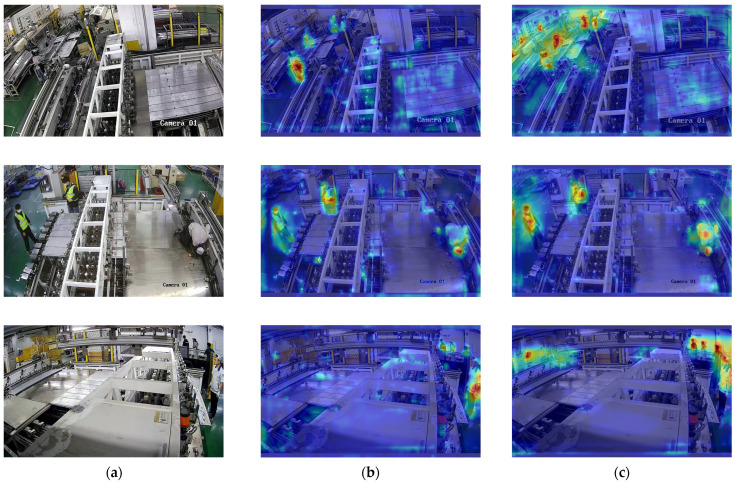
Comparison of the heatmaps between the baseline and our model. (**a**) Original images; (**b**) baseline; (**c**) our model.

The images used for analysis are selected from the industrial site and are not utilized during the training phase of the object detection model. As shown in Figure 11, the heatmaps for small targets are notably less intense, suggesting a weaker emphasis on these objects, and the heatmap in the first row of Figure 11b even overlooks targets located at a distance. In contrast, our improved YOLOv8n model shows significant enhancements in these areas. The heatmaps generated by the improved model exhibit a noticeable increase in intensity for both small and distant targets, indicating a stronger focus. These visual analyses clearly demonstrate the advantages of our improved YOLOv8n model and confirm that the diverse dataset we constructed, along with the data augmentation strategies, effectively mitigated the issue of overfitting and enhanced the model’s generalization performance.

### 4.4. Experiments on Tracking

In the object tracking step, the target object is the person class. We utilize the Reid model pre-trained on the Market-1501 dataset [50], which contains a vast array of pedestrian images captured from various camera perspectives and lighting conditions, providing the model with rich features to learn how to recognize and distinguish different individuals. Therefore, this paper opts not to retrain the Reid model.

**Figure 12:**
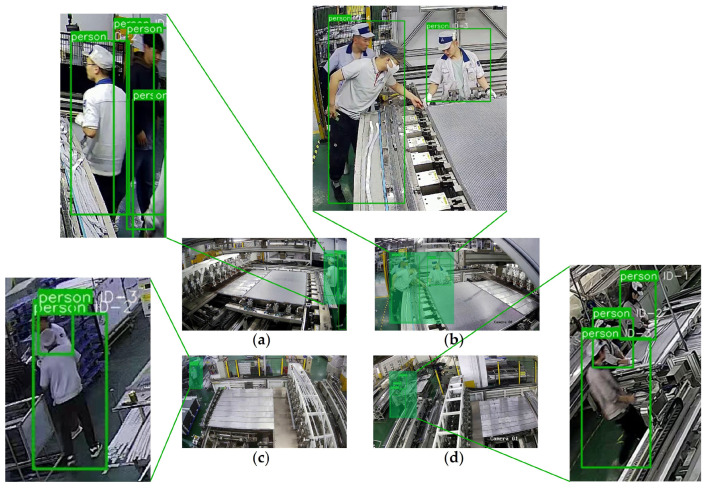
Tracking visualization of four scenes. (**a**–**d**) Scenes 1–4. (**a**,**b**) show moments of tracking failure, including false positives and false negatives. (**c**,**d**) Illustrate moments of stable tracking when the monitoring is near a top-view perspective.

We select four challenging video segments totaling 1250 frames, which includes scenarios with multiple overlapping, varying distances of targets, and other complex situations. We then label these frames using X-AnyLabeling [51] to create the tracking testing set. For detailed experimental scenes and tracking result evaluations, please refer to Figure 12 and Table 7.

Table 7 shows that DeepSORT achieves excellent tracking performance in scenes 3 and 4, even in cases where targets are distant from the camera, and occlusions and overlaps occur. One aspect is attributed to the relatively short duration of occlusion periods for individuals in the frame, naturally leading to fewer occurrences of false positives and false negatives. Another aspect is the placement strategy of surveillance cameras, further alleviating the impact of overlapping targets. Positioning the camera at or near a top-view perspective can greatly enhance the performance of the object tracking algorithm [52]. In contrast, in scenes 1 and 2, DeepSORT exhibits low MOTA and MOTP values. It is mainly attributed to a significant number of false positives and false negatives occurring when individuals overlap or new members enter the scenes, as illustrated in Figure 12a,b. In our setup, guided by the dress judgment criterion, we label a target as a person class only when both the head and upper body are visible in the frame. However, when individuals enter the frame with partial body visibility, the model could produce false positives.

In summary, we observe that even in various challenging scenes, the minimum achieved MOTA and MOTP values, respectively, reach 67% and 75%. The current evaluation results demonstrate that the algorithm’s possession of the fundamental person tracking capability in this industrial scene. Additionally, the overall detection and tracking speed exceeds 55 FPS, meeting the requirements for real-time monitoring.

### 4.5. Overall Performance Comparison

**Figure 13:**
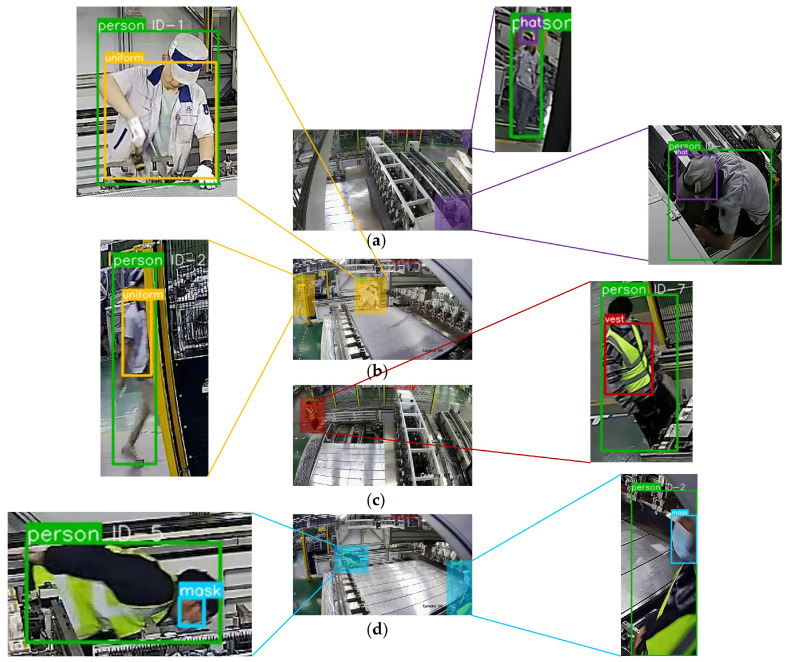
Monitoring snapshots of different dress classes. (**a**–**d**) Hat, uniform, vest, and mask.

**Figure 14:**
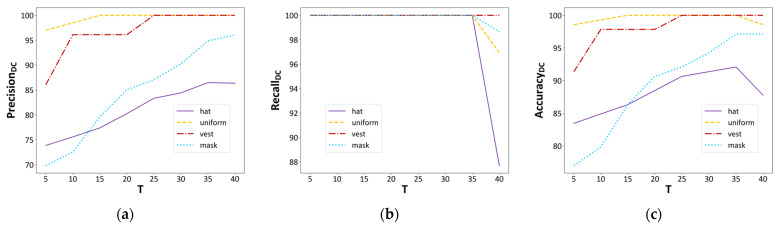
The overall judgment performance of our instance-level method. (**a**) ${P\mathrm{recision}}_{\mathrm{DC}}$; (**b**) ${R\mathrm{ecall}}_{\mathrm{DC}}$; (**c**) ${A\mathrm{ccuracy}}_{\mathrm{DC}}$. When G is set to 40, as T increases, the evaluation metrics for judgment of the four dress-related objects steadily improve. However, when T is set too high and approaches the value of G, performance will decline.

Our final dress code monitoring method is evaluated using videos that are not included in the model training phase. These videos, obtained from multiple on-site surveillance cameras, total approximately 4 min and comprise 5750 frames, encompassing various lighting conditions and different personnel. To demonstrate the generalizability of the experiments, the individuals in the videos include a range of poses such as squatting, turning around, and routine work activities. Additionally, to ensure fairness and comparability of the final experimental results, we carefully balance the duration of diverse dress conditions, whether the dress code is adhered to or violated, throughout the videos. Specific experimental scenes and outcomes are depicted in Figure 13 and Figure 14 and Table 8, using the parameter G = 40 as an example.

As shown in Table 8, when only using the original YOLOv8n model for dress judgment, the ${\mathrm{recall}}_{\mathrm{DC}}$ reaches close to 100%, indicating that under the bbox-level judgment criterion, the model can identify the majority of violations. However, except for the vest category, the ${\mathrm{precision}}_{\mathrm{DC}}$ of other categories are unsatisfactory, reflecting a major flaw in this judgment criterion, i.e., erroneously identifying many compliant dress situations as non-compliant. When we replace the original model with our improved YOLOv8n model for bbox-level judgment, all evaluation metrics show some improvement, especially a significant increase in ${\mathrm{precision}}_{\mathrm{DC}}$ for the uniform category, which increases by 3.8%. Nevertheless, these improvements still fall short of the requirements for dress judgment in practical applications, indicating that relying solely on object detection models is insufficient for dress monitoring tasks.

By combining the improved YOLOv8n model with DeepSORT and our dress code judgment criterion, we achieve instance-level dress judgment across multi-frames, and this integration leads to improvements in all evaluation metrics. Specifically, the ${\mathrm{recall}}_{\mathrm{DC}}$ for all dress categories reaches 100%, suggesting that the model can identify all instances of dress violations, while the ${\;\mathrm{precision}}_{\mathrm{DC}}$ and ${\mathrm{accuracy}}_{\mathrm{DC}}$ also improve significantly. Additionally, as depicted in Figure 14, we observe that as the threshold parameter T increases, the model’s ${\mathrm{precision}}_{\mathrm{DC}}$ and ${\mathrm{accuracy}}_{\mathrm{DC}}$ show an upward trend before T = 35, indicating a gradual decrease in false alarms. However, as T continues to increase and approaches the value of G, the ${\mathrm{recall}}_{\mathrm{DC}}$ value sharply declines, adversely affecting the overall judgment performance of our method. Therefore, in this application scenario, T = 35 could be the optimal choice when G is set to 40.

These results highlight the advantages of the instance-level judgment method for dress code monitoring tasks over the traditional bbox-level method. Nevertheless, as illustrated in Figure 8, occasional misjudgments still occur regarding the detection of hats and masks. This is primarily due to personnel being positioned at a greater distance from the camera, where such small objects become even less distinguishable, resulting in instances where the system recognizes the person but fails to detect their dresses, even when properly worn. Despite these challenges, the proposed method remains effective and well-suited for practical on-site deployment.

It should be noted that, in practical application scenarios, the parameters T and G can be adjusted flexibly to optimize monitoring effectiveness based on specific needs. For example, modifications can be made according to the strictness of dress code regulations. Increasing the T value appropriately can improve the accuracy of dress code judgments, while raising the G value allows for more lenient monitoring.

## 5. Conclusions

This paper proposes a novel method for dress code monitoring, which divides the task into three steps. First, the YOLOv8n object detection model is improved by introducing the FPF structure, RFAConv, and FLatten module, which significantly improves detection performance while maintaining the lightweight model. Through multidimensional comparative experiments, we thoroughly verify the performance improvements brought by these enhancements. Next, DeepSORT is integrated to obtain instance information across multi-frames. Finally, the instance-level judgment criterion is adopted to conduct real-scene dress code monitoring. Our method demonstrates significant effectiveness and advancement compared to traditional bbox-level methods that rely solely on object detection. Nevertheless, the method still encounters some misjudgment issues for small targets located at a greater distance from the camera. Future research will focus on exploring more advanced structures and effective attention mechanisms to improve detection performance for small targets. Additionally, we plan to investigate alternative tracking algorithms and expand the dataset to cover a wider range of industrial contexts beyond the current indoor settings discussed in this study. We anticipate that the proposed method will be widely applicable in various dress monitoring scenarios, contributing to technological advancements in this field.

## Figures and Tables

**Table 1:** Definition and description of ${\mathrm{TP}}_{\mathrm{DC}}$, ${\mathrm{FP}}_{\mathrm{DC}}$, ${\mathrm{TN}}_{\mathrm{DC}}$, and ${\mathrm{FN}}_{\mathrm{DC}}$.

Definition	Description
${\mathrm{TP}}_{\mathrm{DC}}$	Throughout G frames being tracked, the person violates the dress code, as predicted by the model.
${\mathrm{FP}}_{\mathrm{DC}}$	Throughout G frames being tracked, the person adheres to the dress code, but the model predicts it as non-compliance.
${\mathrm{TN}}_{\mathrm{DC}}$	Throughout G frames being tracked, the person adheres to the dress code, as predicted by the model.
${\mathrm{FN}}_{\mathrm{DC}}$	Throughout G frames being tracked, the person violates the dress code, but the model predicts it as compliance.

**Table 2:** Detection results of YOLOv8n, YOLOv8n-P2, and YOLOv8n-FPF. The bold data in the table indicate the best results, and this applies to the following tables as well.

Models	${\mathrm{Precision}}_{\mathrm{Det}}$ (%)	${\mathrm{Recall}}_{\mathrm{Det}}$ (%)	${\mathrm{mAP}}_{\mathrm{Det}}0.5$ (%)	${\mathrm{mAP}}_{\mathrm{Det}}0.5:0.95$ (%)	Model Size (MB)	GFLOPs	FPS
YOLOv8n	89.3	79.9	86.9	53.9	6.2	**8.2**	**218**
YOLOv8n-P2	89.3	83	89.7	56.5	6.3	12.4	171
YOLOv8n-FPF	**90.4**	**84.2**	**90.2**	**57.3**	**5.4**	12.1	174

**Table 3:** Comparative experimental results for different insertion locations of the FLatten module.

Models	${\mathrm{Precision}}_{\mathrm{Det}}$ (%)	${\mathrm{Recall}}_{\mathrm{Det}}$ (%)	${\mathrm{mAP}}_{\mathrm{Det}}$0.5 (%)	${\mathrm{mAP}}_{\mathrm{Det}}$0.5:0.95 (%)
Baseline	89.4	**86.1**	90.7	57.8
+ Location 1	90.3	84	90.6	57.7
+ Location 2	90.8	83.6	90.4	57.7
**+ Location 3**	90.7	84.9	**90.8**	**58.2**
+ Location 4	**91.8**	82.1	90.4	57.3
+ Location 5	89.8	85.2	90.6	57.7

**Table 4:** Comparison of detection accuracy of each dress class between the original YOLOv8n and our model.

Models	Person (%)	Uniform (%)	Vest (%)	Hat (%)	Mask (%)	${\mathrm{mAP}}_{\mathrm{Det}}$0.5 (%)
YOLOv8n	89.3	87.4	96.6	77.2	81.5	86.9
Ours	**91.6**	**89.6**	**96.8**	**85.9**	**89.5**	**90.8**

**Table 5:** Comparative experiments of different models.

Models	${\mathrm{mAP}}_{\mathrm{Det}}$0.5 (%)	${\mathrm{mAP}}_{\mathrm{Det}}$0.5:0.95 (%)	Model Size (MB)	GFLOPs	FPS
SSD [42]	72.1	33.2	97.1	275	26
Faster-RCNN [30]	69.8	30.7	547.1	401.8	16
YOLOv5n [43]	84.5	46	**3.8**	**4.1**	**294**
YOLOv5s [43]	87.4	49.9	14.4	15.8	227
YOLOv7-tiny [44]	88.7	50.7	12.3	13.2	222
YOLOv8n [5]	86.9	53.9	6.2	8.2	218
YOLOv8s [5]	89.3	57.8	22.5	28.7	133
YOLOv9t [45]	85.5	53.4	6	10.7	92
YOLOv10n [46]	85.5	51.5	5.8	6.5	204
GOLD-YOLO [47]	84.2	47.1	49.5	12.5	147
RT-DETR-Resnet18 [48]	90.4	57.4	322.3	60.9	57
**Ours**	**90.8**	**58.2**	5.7	12.4	100

**Table 6:** Ablation study results.

Baseline	FPF	RFAConv	FLatten	${\mathrm{mAP}}_{\mathrm{Det}}0.5$ (%)	${\mathrm{mAP}}_{\mathrm{Det}}$0.5:0.95 (%)	Model Size (MB)	GFLOPs	FPS
YOLOv8n				86.9	53.9	6.2	**8.2**	**218**
	√			90.2	57.3	**5.4**	12.1	174
		√		87.7	55.2	6.4	8.4	152
			√	87.7	54.6	6.5	**8.2**	211
	√	√		90.7	57.8	5.5	12.1	110
	√	√	√	**90.8**	**58.2**	5.7	12.4	100

**Table 7:** Tracking performance of four scenes.

Model	Scene	MOTA (%)	MOTP (%)	FPS
Improved YOLOv8n + DeepSORT	1	67	75	56.5
	2	71	80	55.6
	3	92	82	61.3
	4	93	82	56.8

**Table 8:** Performance comparison of the dress code judgment methods.

Models	Judgment Criterion	Category	${\mathrm{Precision}}_{\mathrm{DC}}$ (%)	${\mathrm{Recall}}_{\mathrm{DC}}$ (%)	${\mathrm{Accuracy}}_{\mathrm{DC}}$ (%)
YOLOv8n	Bbox-level	hat	78.4	99.1	86.5
		uniform	84.1	99.7	90.8
		vest	96	**100**	97.8
		mask	78.4	99.9	85.6
Improved YOLOv8n	Bbox-level	hat	81.1	99	88.4
		uniform	87.9	99.9	93.4
		vest	96.6	**100**	98.1
		mask	79.5	99.9	86.5
Improved YOLOv8n + DeepSORT	Instance-level (T = **35**, G = 40)	hat	**86.7**	**100**	**92.8**
		uniform	**100**	**100**	**99.4**
		vest	**100**	**100**	**100**
		mask	**94.9**	**100**	**99.1**

T: The threshold number of hazardous boxes; G: the tracking frame cycle for each instance’s dress judgment.

## Data Availability

Data are not publicly available and can be obtained by contacting the corresponding author if necessary.

## References

[1] Na Z., Zechuan Y., You H., Xiaoan B., Yifan S. Personnel Dress Code Detection Algorithm Based on Convolutional Neural Network Cascade. Proceedings of the 2020 2nd International Conference on Machine Learning, Big Data and Business Intelligence (MLBDBI).

[2] Zhang Q., Pei Z., Guo R., Zhang H., Kong W., Lu J., Liu X. (2022). An Automated Detection Approach of Protective Equipment Donning for Medical Staff under COVID-19 Using Deep Learning. Comput. Model. Eng. Sci..

[3] Zhou Z., Zhou C., Pan A., Zhang F., Dong C., Liu X., Zhai X., Wang H. (2023). A Kitchen Standard Dress Detection Method Based on the YOLOv5s Embedded Model. Appl. Sci..

[4] Lin B. (2024). Safety Helmet Detection Based on Improved YOLOv8. IEEE Access.

[5] Ultralytics/Ultralytics: YOLOv8. https://github.com/ultralytics/ultralytics.

[6] Ma W., Guan Z., Wang X., Yang C., Cao J. (2023). YOLO-FL: A Target Detection Algorithm for Reflective Clothing Wearing Inspection. Displays.

[7] Lyu Y., Yang X., Guan A., Wang J., Dai L. (2024). Construction Personnel Dress Code Detection Based on YOLO Framework. CAAI Trans. Intell. Technol..

[8] An Q., Xu Y., Yu J., Tang M., Liu T., Xu F. (2023). Research on Safety Helmet Detection Algorithm Based on Improved YOLOv5s. Sensors.

[9] Chen L., Mao Y., Zhang H., Luan S. Improved the Detection Algorithm of Safety Helmet Wearing Based on YOLOv8. Proceedings of the 2023 3rd International Conference on Electronic Information Engineering and Computer Communication (EIECC).

[10] Li J., Li Y., Villaverde J.F., Chen X., Zhang X. (2023). A Safety Wearing Helmet Detection Method Using Deep Leaning Approach. J. Opt..

[11] Agarwal D., Gupta P., Eapen N.G. A Framework for Dress Code Monitoring System Using Transfer Learning from Pre-Trained YOLOv4 Model. Proceedings of the 2023 11th International Conference on Emerging Trends in Engineering & Technology—Signal and Information Processing (ICETET—SIP).

[12] LI Q., Wei J. (2023). Lightweight Real-Time Detection Method for Dress Code of Anti-Static Equipment. Acad. J. Comput. Inf. Sci..

[13] Wojke N., Bewley A., Paulus D. Simple Online and Realtime Tracking with a Deep Association Metric. Proceedings of the 2017 IEEE International Conference on Image Processing (ICIP).

[14] Zhang X., Liu C., Yang D., Song T., Ye Y., Li K., Song Y. (2023). RFAConv: Innovating Spatial Attention and Standard Convolutional Operation 2023. arXiv.

[15] Han D., Pan X., Han Y., Song S., Huang G. (2023). FLatten Transformer: Vision Transformer Using Focused Linear Attention 2023. arXiv.

[16] Chen H., Zhou G., Jiang H. (2023). Student Behavior Detection in the Classroom Based on Improved YOLOv8. Sensors.

[17] Cheng G., Yuan X., Yao X., Yan K., Zeng Q., Xie X., Han J. (2023). Towards Large-Scale Small Object Detection: Survey and Benchmarks. IEEE Trans. Pattern Anal. Mach. Intell..

[18] Han H., Zhu F., Zhu B., Wu H. (2023). Target Detection of Remote Sensing Image Based on an Improved YOLOv5. IEEE Geosci. Remote Sens. Lett..

[19] Guo B., Ling S., Tan H., Wang S., Wu C., Yang D. (2023). Detection of the Grassland Weed Phlomoides Umbrosa Using Multi-Source Imagery and an Improved YOLOv8 Network. Agronomy.

[20] Zhu X., Cheng D., Zhang Z., Lin S., Dai J. An Empirical Study of Spatial Attention Mechanisms in Deep Networks. Proceedings of the IEEE/CVF International Conference on Computer Vision.

[21] Woo S., Park J., Lee J.-Y., Kweon I.S. CBAM: Convolutional Block Attention Module. Proceedings of the European Conference on Computer Vision (ECCV).

[22] Hou Q., Zhou D., Feng J. Coordinate Attention for Efficient Mobile Network Design. Proceedings of the IEEE/CVF Conference on Computer Vision and Pattern Recognition.

[23] Khan S., Naseer M., Hayat M., Zamir S.W., Khan F.S., Shah M. (2022). Transformers in Vision: A Survey. ACM Comput. Surv..

[24] Choromanski K., Likhosherstov V., Dohan D., Song X., Gane A., Sarlos T., Hawkins P., Davis J., Mohiuddin A., Kaiser L. Rethinking Attention with Performers. Proceedings of the International Conference on Learning Representations.

[25] Shen Z., Zhang M., Zhao H., Yi S., Li H. Efficient Attention: Attention With Linear Complexities. Proceedings of the IEEE/CVF Winter Conference on Applications of Computer Vision.

[26] Li H., Li J., Wei H., Liu Z., Zhan Z., Ren Q. (2024). Slim-Neck by GSConv: A Better Design Paradigm of Detector Architectures for Autonomous Vehicles. J. Real-Time Image Proc..

[27] Xiang C., Yin D., Song F., Yu Z., Jian X., Gong H. (2024). A Fast and Robust Safety Helmet Network Based on a Mutilscale Swin Transformer. Buildings.

[28] Lee J.-Y., Choi W.-S., Choi S.-H. (2023). Verification and Performance Comparison of CNN-Based Algorithms for Two-Step Helmet-Wearing Detection. Expert Syst. Appl..

[29] Tan M., Le Q. EfficientNet: Rethinking Model Scaling for Convolutional Neural Networks. Proceedings of the 36th International Conference on Machine Learning, PMLR.

[30] Ren S., He K., Girshick R., Sun J. (2017). Faster R-CNN: Towards Real-Time Object Detection with Region Proposal Networks. IEEE Trans. Pattern Anal. Mach. Intell..

[31] Lin T.-Y., Goyal P., Girshick R., He K., Dollar P. Focal Loss for Dense Object Detection. Proceedings of the IEEE International Conference on Computer Vision.

[32] Jia W., Xu S., Liang Z., Zhao Y., Min H., Li S., Yu Y. (2021). Real-time Automatic Helmet Detection of Motorcyclists in Urban Traffic Using Improved YOLOv5 Detector. IET Image Process..

[33] Wei C., Yang X. Dress Code Surveillance at Power Grid Construction Site via Object Detection. Proceedings of the 2021 3rd International Conference on Electrical Engineering and Control Technologies (CEECT).

[34] Jadhav C., Ansari N. Realtime Safety Helmet Detection Using Deep Learning. Proceedings of the 2024 5th International Conference for Emerging Technology (INCET).

[35] Ciaparrone G., Sánchez F.L., Tabik S., Troiano L., Tagliaferri R., Herrera F. (2020). Deep Learning in Video Multi-Object Tracking: A Survey. Neurocomputing.

[36] Bewley A., Ge Z., Ott L., Ramos F., Upcroft B. 16.0Simple Online and Realtime Tracking. Proceedings of the 2016 IEEE International Conference on Image Processing (ICIP).

[37] Kalman R.E. (1960). A New Approach to Linear Filtering and Prediction Problems. J. Basic Eng..

[38] Kuhn H.W. (1955). The Hungarian Method for the Assignment Problem. Nav. Res. Logist. Q..

[39] Kumar S., Vishal, Sharma P., Pal N. Object Tracking and Counting in a Zone Using YOLOv4, DeepSORT and TensorFlow. Proceedings of the 2021 International Conference on Artificial Intelligence and Smart Systems (ICAIS).

[40] Kaggle Mask Dataset. https://www.kaggle.com/datasets/andrewmvd/face-mask-detection.

[41] Milan A., Leal-Taixe L., Reid I., Roth S., Schindler K. (2016). MOT16: A Benchmark for Multi-Object Tracking 2016. arXiv.

[42] Liu W., Anguelov D., Erhan D., Szegedy C., Reed S., Fu C.-Y., Berg A.C. SSD: Single Shot MultiBox Detector. Proceedings of the ECCV.

[43] Ultralytics/Yolov5: YOLOv5. https://github.com/ultralytics/yolov5.

[44] Wang C.-Y., Bochkovskiy A., Liao H.-Y.M. YOLOv7: Trainable Bag-of-Freebies Sets New State-of-the-Art for Real-Time Object Detectors. Proceedings of the 2023 IEEE/CVF Conference on Computer Vision and Pattern Recognition (CVPR).

[45] Wang C.-Y., Yeh I.-H., Liao H.-Y.M. (2024). YOLOv9: Learning What You Want to Learn Using Programmable Gradient Information 2024. arXiv.

[46] Wang A., Chen H., Liu L., Chen K., Lin Z., Han J., Ding G. (2024). YOLOv10: Real-Time End-to-End Object Detection 2024. arXiv.

[47] Wang C., Nie W.H.Y., Guo J., Liu C., Han K., Wang Y. Gold-YOLO: Efficient Object Detector via Gather-and-Distribute Mechanism. Proceedings of the Advances in Neural Information Processing Systems (NeurIPS).

[48] Zhao Y., Lv W., Xu S., Wei J., Wang G., Dang Q., Liu Y., Chen J. DETRs Beat YOLOs on Real-Time Object Detection. Proceedings of the IEEE/CVF Conference on Computer Vision and Pattern Recognition (CVPR).

[49] Selvaraju R.R., Cogswell M., Das A., Vedantam R., Parikh D., Batra D. Grad-CAM: Visual Explanations from Deep Networks via Gradient-Based Localization. Proceedings of the IEEE International Conference on Computer Vision.

[50] Zheng L., Shen L., Tian L., Wang S., Wang J., Tian Q. Scalable Person Re-Identification: A Benchmark. Proceedings of the 2015 IEEE International Conference on Computer Vision (ICCV).

[51] CVHub520/X-AnyLabeling. https://github.com/CVHub520/X-AnyLabeling.

[52] Sharma N., Baral S., Paing M.P., Chawuthai R. (2023). Parking Time Violation Tracking Using YOLOv8 and Tracking Algorithms. Sensors.

